# Association between periventricular hyperintensity or deep white matter hyperintensity and outcomes of patients with ischemic stroke

**DOI:** 10.3389/fneur.2025.1583318

**Published:** 2025-06-25

**Authors:** Hongwei Ren, Du Wang, Xing Wang, Jinlong Wu, Junli Huang, Honghua Jin, Chengfang Huang, Lei Lei

**Affiliations:** ^1^Department of Medical Imaging, Tianyou Hospital Affiliated to Wuhan University of Science and Technology, Wuhan, China; ^2^Department of Neurology, Tianyou Hospital Affiliated to Wuhan University of Science and Technology, Wuhan, China

**Keywords:** ischemic stroke, periventricular hyperintensity, deep white matter hyperintensity, functional outcome, recurrence

## Abstract

**Introduction:**

This study investigates the association between white matter hyperintensities (WMH), specifically periventricular hyperintensity (PVH) and deep white matter hyperintensity (DWMH), and the prognosis and recurrence risk in patients with ischemic stroke.

**Methods:**

A retrospective analysis was conducted on 278 patients with acute ischemic stroke, including 112 with PVH and 166 with DWMH. Key outcome measures included functional outcome (modified Rankin Scale score), mortality, neurological recovery, and stroke recurrence at 3, 6, and 12 months after treatment.

**Results:**

Severe PVH was significantly associated with unfavorable functional outcomes at 3, 6, and 12 months, while severe DWMH was only linked to unfavorable outcomes at 3 months. Mild PVH, but not DWMH, was associated with neurological recovery. Both higher PVH and DWMH were significantly correlated with stroke recurrence, with PVH showing an association at 12 months and DWMH at all time points. Severe PVH, but not DWMH, was also associated with stroke-related deaths.

**Discussion:**

These findings highlight the significant prognostic value of PVH and DWMH subtypes in ischemic stroke, suggesting their potential utility in predicting long-term functional outcomes, recurrence, and mortality.

## Introduction

Ischemic stroke refers to paralysis and consciousness disorders caused by cerebral thrombosis or cerebral thrombosis, which leads to cerebral infarction and cerebral artery blockage (1). In ischemic stroke, when a blood clot or embolism forms within the affected blood vessel, the brain tissue supplied by that vessel will suffer infarction, local edema, and congestion of the surrounding tissues. Over the course of several hours to several days, the affected area will swell, leading to ischemic necrosis and softening. Then, the necrotic tissue gradually liquefies to form cystic cavities, and eventually may leave a yellow atrophic scar (2). The prognosis of stroke varies from individual to individual. Even for cases of stroke that occur in the same area and have similar lesion sizes, the recovery outcomes can still be quite different (3). In addition, the recurrence risks of different types of ischemic strokes vary. For instance, in lacunar strokes, the recurrent lacunar lesions are the most common cause of subcortical dementia; while in cardiogenic embolic strokes, this factor is the most important predictor of mortality (4). Therefore, it is of great clinical significance to explore the factors influencing the prognosis and recurrence of stroke. Existing research results have found that age, gender, severity of stroke, and vascular risk factors may affect the prognosis of stroke, and different studies have reached different conclusions (5, 6).

With the gradual development of brain imaging technology, researchers have found that abnormal signal changes that occur when the white matter in the lateral ventricles or subcortical regions is symmetrically distributed are more common in the elderly or stroke patients. Due to their high signal intensity in MRI’s T2 and FLAIR sequences, this phenomenon is called white matter hyperintensity (WMH). Imaging and pathological studies have shown that the abnormal signals in the white matter are pathologically manifested as gliosis, myelin degeneration, axonal loss, small-scale infarction, and expansion of the perivascular space. Therefore, they are regarded as one of the important imaging indicators of cerebellar vascular diseases. According to the research, this vascular-originated WMH is closely related to clinical manifestations such as cognitive decline, increased risk of stroke, gait instability, falls, and depression (7, 8).

According to the location of WMH, WMH is divided into periventricular hyperintensity (PVH) and deep white matter hyperintensity (DWMH). Studies have shown that there are differences in pathology between PVH and DWMH. Under the microscope, PVH presents demyelinating lesions, accompanied by subependymal glial proliferation and loss of ependymal continuity, and is not an ischemic lesion, while DWMH mainly presents as small cystic infarctions and belongs to ischemic lesions. This suggests that the pathogenesis of WMH in different brain regions may vary. Such differences are also reflected in different clinical manifestations. The Rotterdam study found that the cognitive function impairment was more closely related to PVH, while some studies on depression have suggested that DWMH had a stronger correlation with it (9, 10). In terms of correlation with the prognosis of stroke, according to pathological findings, it seems that ischemic lesions of DWMH are more closely related to the prognosis of stroke. However, some previous studies have reached the opposite conclusion, suggesting that PVH has a stronger correlation with the prognosis of stroke than DWMH. Therefore, the difference between PVH and DWMH in predicting the prognosis of acute ischemic stroke is also the focus of this study (11).

This study aimed to study the impact of correlation studies on the WMH in MRI of patients with ischemic stroke, as well as their long-term prognosis and recurrence risk. The discussion was conducted separately based on the prognosis conditions at different time points and the differences between PVH and DWMH, in order to explore whether there are differences in functional prognosis among stroke patients with different distribution characteristics or severity of WMH.

## Methods

### Research object

**Figure 1:**
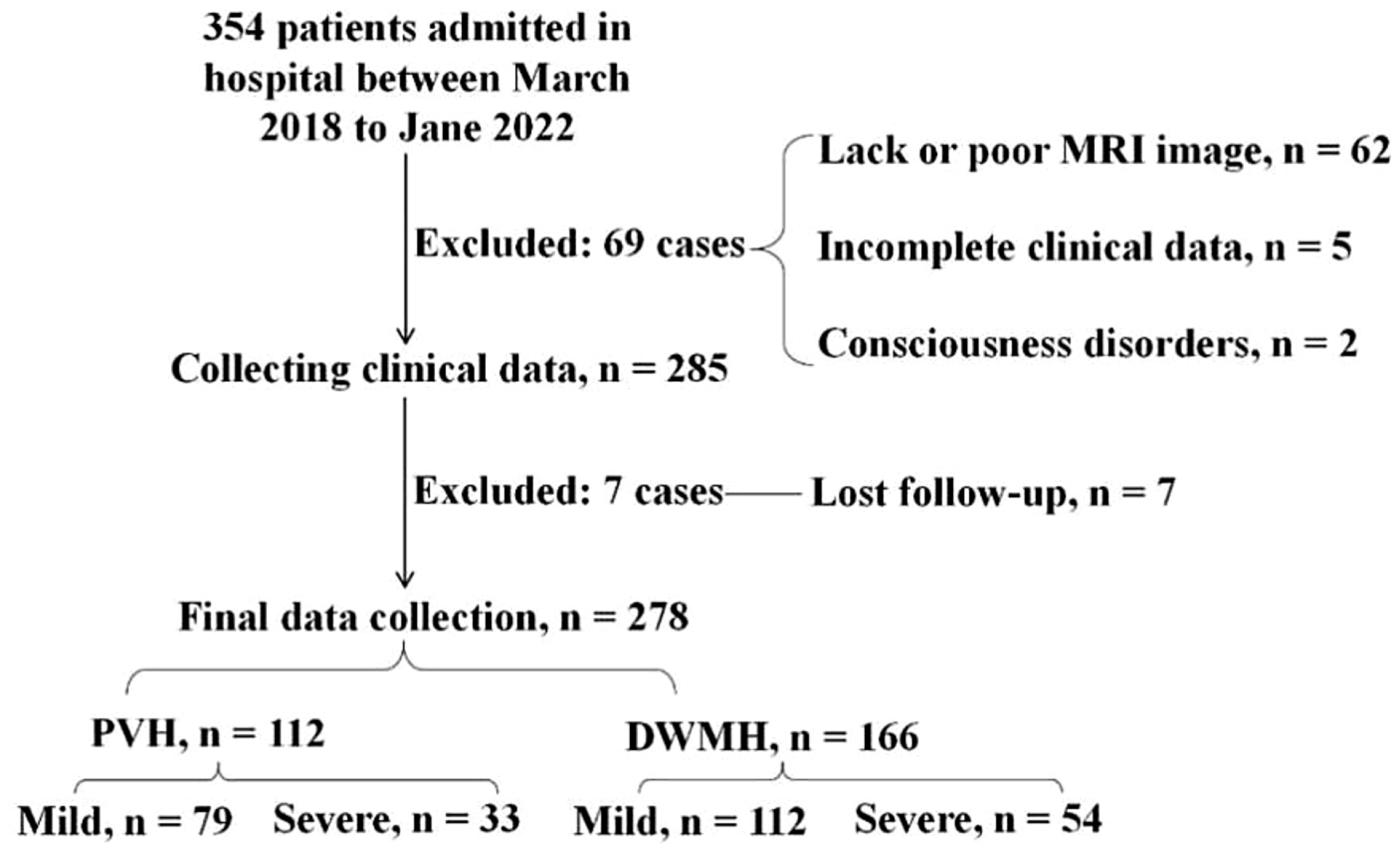
Inclusion of research objects.

A retrospective study was performed based on general data from 278 cases of acute ischemic stroke patients admitted to the Tianyou Hospital Affiliated to Wuhan University of Science and Technology from March 2018 to Jane 2022. This study was approved by the Ethics Committee of the Tianyou Hospital Affiliated to Wuhan University of Science and Technology. Inclusion criteria: (1) all patients were in line with the relevant diagnostic criteria set out in the Chinese Cerebrovascular Disease Management Consensus (2015) and confirmed by CT and head MRI; (2) time of onset < 30 days; (3) aged 41–78 years old; (4) the modified Rankin Scale (mRS) score at discharge was 0–5 points; (5) all patients and their families were informed about this study and signed informed consent forms. Patients with the following conditions were excluded from the study: (1) severe heart, liver, and renal dysfunction; (2) previous acute ischemic stroke, cerebral infarction with hemorrhage, and intracranial hemorrhage; (3) acute and chronic infectious diseases, hematological diseases, autoimmune diseases, and tumors; (4) history of active bleeding or head trauma; (5) mental disorders, consciousness disorders, and dementia; (6) expected survival time less than 2 years; (7) poor MRI image quality which lacks sequences to assess the degree of vascular stenosis or the severity of white matter lesions; (8) incomplete clinical data and lost follow-up data; (9) being on other clinical trials. Total 354 patients were admitted in our hospital. After excluding 62 cases lacking MRI image of having poor MRI image quality, 5 cases with incomplete data, 2 cases with consciousness disorders, and 7 lost follow-up, 278 cases were included (Figure 1). This study was approved by the Ethics Committee of the Tianyou Hospital Affiliated to Wuhan University of Science and Technology. All participants provided written informed consent before enrollment, and the study was conducted in accordance with the Declaration of Helsinki.

### Assessment of WMLs

The evaluation of WMH was mainly based on FLAIR sequence or T2-weighted sequence of MRI (Siemens 3.0 T Magnetic Resonance Imaging System, Siemens, Germany). FLAIR sequence was preferred during evaluation, and T2-weighted sequence was used for patients with no or poor image quality of FLAIR sequence. Fazekas score (12) was used to score PVH and DWMH. The scoring criteria of PVH are: 0 score for no white matter high signal, 1 point for cap-like abnormally high signals in the frontal or occipital corners of the lateral ventricles, 2 points for lunar halo-like abnormally high signal around the lateral ventricle, 3 points for irregular periventricular lesions extended to deep white matter. The score criteria of DWMH are: 0 point for no white matter high signal, 1 point for spot-like abnormal high signal intensity in subcortical white matter, 2 points for plaque-like abnormal high signal intensity (there was a tendency of fusion between the lesions), 3 points for the scale of the large continuous lesion area. Both PVH and DWMH were classified as mild on a scale of 0–2 and severe on a scale of 3.

### Outcome measures

The primary outcome measure was functional outcome, defined as the modified Rankin Scale (mRS) score of 0–6 (higher scores indicated more disability) (13) at 3, 6, and 12 months after treatment. The score of 0–2 indicated favorable functional outcome, and score of 3–6 indicated unfavorable functional outcome. Mortality, early neurological recovery, and recurrence at 3, 6, and 12 months after treatment were secondary clinical outcome measures. Early neurological recovery was defined as a National Institutes of Health Stroke Scale (NIHSS) 22 score of 0 or 1 at 24 h after symptom onset or an improvement in NIHSS at 24 h of at least 8 points relative to baseline NIHSS (14).

### Statistical analysis

We analyzed the association between PVH or DWMH and clinical data or outcome measures of patients with ischemic stroke using Student’s t test or chi-square test. NIHSS scores are expressed as median and interquartile range, while other continuous values are expressed as mean ± standard deviation. Counting data are expressed as n (%). *p* < 0.05 indicated statistical significance. All figures and statistical analyses were done using GraphPad software version 8.

## Results

### Comparison of baseline data between mild and severe group of PVH

Table 1 summarized the baseline data of PVH patients. The average age of PVH patients was (65.34 ± 11.03) years, with the age of the severe group being significantly higher than that of the mild group. There were 78 male patients (69.64%) and 34 female patients (30.36%). There were 58 cases of hemorrhagic ischemic stroke and 54 cases of thrombotic ischemic stroke. The number of cases with hypertension, diabetes, hyperlipidemia, stroke or transient ischemic attack, coronary heart disease and atrial fibrillation were 62, 22, 64, 16, 19 and 39, respectively. There were no differences in gender, acute ischemic stroke subtypes, hypertension and smoking between the two groups of PVH patients. In the PVH group, the proportion of patients with diabetes, hyperlipidemia, stroke or transient ischemic attack, coronary heart disease and atrial fibrillation in the severe group was significantly higher than that in the mild group, respectively (*p* = 0.0017, *p* = 0.0014, *p* = 0.0046, *p* = 0.0068 and *p* = 0.0089).

**Table 1:** Comparison of baseline data between mild and severe group of PVH.

Characteristic	Total *n* = 112	Mild group *n* = 79	Severe group *n* = 33	t/z/*x*^2^ value	*p* value
Age (years,^−^x ± s)	65.34 ± 11.03	62.34 ± 10.14	69.12 ± 14.27	2.885	0.0053
Baseline NIHSS, M (p25, p75)	15 (10, 22)	14 (10, 17)	19 (10, 22)	5.423	<0.001
Gender (*n*, %)					
Males	78 (69.64%)	55 (69.62%)	23 (69.70%)	0.217	0.828
Females	34 (30.36%)	24 (30.38%)	10 (30.30%)		
Acute ischemic stroke subtypes (*n*, %)					
Hemorrhagic ischemic stroke	58 (51.79%)	39 (49.37%)	19 (57.58%)	0.628	0.428
Thrombotic ischemic stroke	54 (48.21%)	40 (50.63%)	14 (42.42%)		
Hypertension (*n*, %)					
Yes	62 (55.36%)	41 (51.90%)	21 (63.64%)	0.931	0.352
No	50 (44.64%)	38 (48.10%)	12 (36.36%)		
Diabetes (*n*, %)					
Yes	22 (19.64%)	9 (11.39%)	13 (39.39%)	3.140	0.0017
No	90 (80.36%)	70 (88.61%)	20 (60.61%)		
Hyperlipidemia (*n*, %)					
Yes	64 (57.14%)	37 (46.84%)	27 (81.82%)	3.201	0.0014
No	48 (42.86%)	42 (53.16%)	6 (18.18%)		
Smoking (*n*, %)					
Yes	48 (42.86%)	31 (39.24%)	17 (51.52%)	0.987	0.324
No	64 (57.14%)	48 (60.76%)	16 (48.48%)		
Stroke or transient ischemic attack (*n*, %)					
Yes	16 (14.29%)	6 (7.59%)	10 (30.30%)	2.835	0.0046
No	96 (85.71%)	73 (92.41%)	23 (69.70%)		
Coronary heart disease (*n*, %)					
Yes	19 (16.96%)	8 (10.13%)	11 (33.33%)	2.707	0.0068
No	93 (83.04%)	71 (89.87%)	22 (66.67%)		
Atrial fibrillation (*n*, %)					
Yes	39 (34.82%)	21 (26.58%)	18 (54.55%)	2.614	0.0089
No	73 (65.18%)	58 (73.42%)	15 (45.45%)		

### Comparison of baseline data between mild and severe group of DWMH

Table 2 revealed the baseline data of patients with DWMH. Among them, there are 86 male patients (51.81%) and 80 female patients (48.19%). There were 86 cases of hemorrhagic ischemic stroke and 80 cases of thrombotic ischemic stroke. The number of cases with hypertension, diabetes, hyperlipidemia, stroke or transient ischemic attack, coronary heart disease, and atrial fibrillation were 75, 31, 77, 27, 24, and 41, respectively. The mean age of patients with DWMH was (63.75 ± 10.83) years, and the age of the severe group was significantly higher than that of the mild group. The proportion of patients with diabetes, hyperlipidemia, stroke or transient ischemic attack, and coronary heart disease in the severe group was significantly higher than that in the mild group. There were no differences in gender, hypertension, acute ischemic stroke subtypes, smoking, and atrial fibrillation between the two groups of patients with PVH (*p* > 0.05).

**Table 2:** Comparison of baseline data between mild and severe group of DWMH.

Characteristic	Total *n* = 166	Mild group *n* = 112	Severe group *n* = 54	t/z/x^2^ value	*p* value
Age (years,^−^x ± s)	63.75 ± 10.83	60.23 ± 10.04	67.84 ± 15.11	3.855	0.0002
Baseline NIHSS, M (p25, p75)	15 (9, 21)	15 (9, 18)	19 (11, 21)	6.742	<0.001
Gender					
Males (*n*, %)	86 (51.81%)	62 (55.36%)	24 (44.44%)	1.152	0.249
Females (*n*, %)	80 (48.19%)	50 (44.64%)	30 (55.56%)		
Acute ischemic stroke subtypes (*n*, %)					
Hemorrhagic ischemic stroke	86 (51.81%)	60 (53.57%)	26 (48.15%)	0.429	0.512
Thrombotic ischemic stroke	80 (48.19%)	52 (46.43%)	28 (51.85%)		
Hypertension (*n*, %)					
Yes	75 (45.18%)	49 (43.75%)	26 (48.15%)	0.367	0.714
No	91 (54.82%)	63 (56.25%)	28 (51.85%)		
Diabetes (*n*, %)					
Yes	31 (18.67%)	14 (12.50%)	17 (31.48%)	2.727	0.0064
No	135 (81.33%)	98 (87.50%)	37 (68.52%)		
Hyperlipidemia (*n*, %)					
Yes	77 (46.39%)	42 (37.50%)	35 (64.81%)	3.140	0.0017
No	89 (53.61%)	70 (62.50%)	19 (35.19%)		
Smoking (*n*, %)					
Yes	83 (50.00%)	52 (46.43%)	31 (57.41%)	1.160	0.246
No	83 (50.00%)	60 (53.57%)	23 (42.59%)		
Stroke or transient ischemic attack (*n*, %)					
Yes	27 (16.27%)	8 (7.14%)	19 (35.19%)	4.334	<0.0001
No	139 (83.73%)	103 (91.96%)	35 (64.81%)		
Coronary heart disease (*n*, %)					
Yes	24 (14.46%)	11 (9.82%)	13 (24.07%)	2.211	0.027
No	142 (85.54%)	101 (90.18%)	41 (75.93%)		
Atrial fibrillation (*n*, %)					
Yes	41 (24.70%)	25 (22.32%)	16 (29.63%)	0.831	0.406
No	125 (75.30%)	87 (77.68%)	38 (70.37%)		

### Association of WMH and mRS score after treatment for 3, 6, 12 months

**Figure 2:**
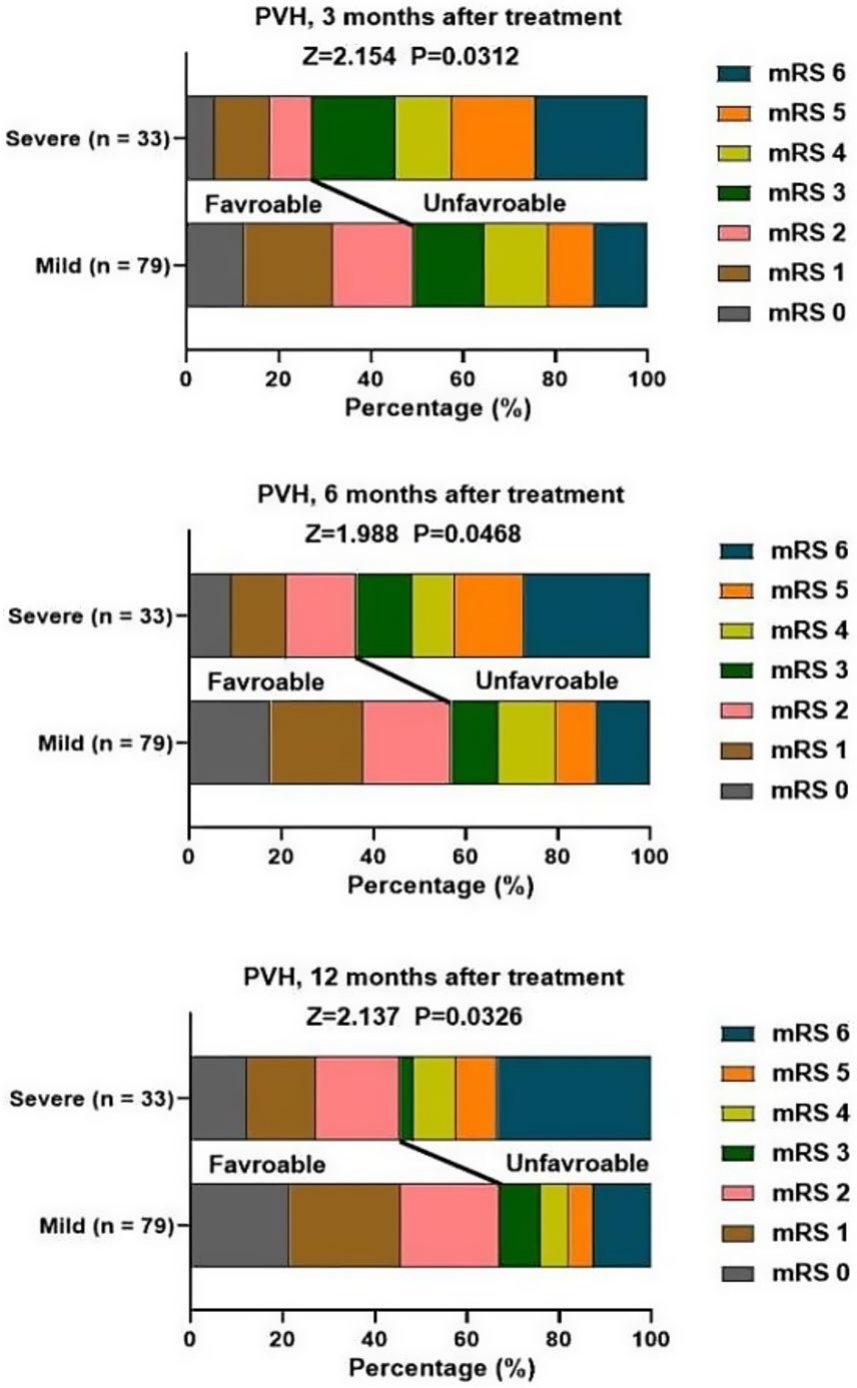
Association of PVH and mRS score at 3, 6, 12 months after treatment.

**Figure 3:**
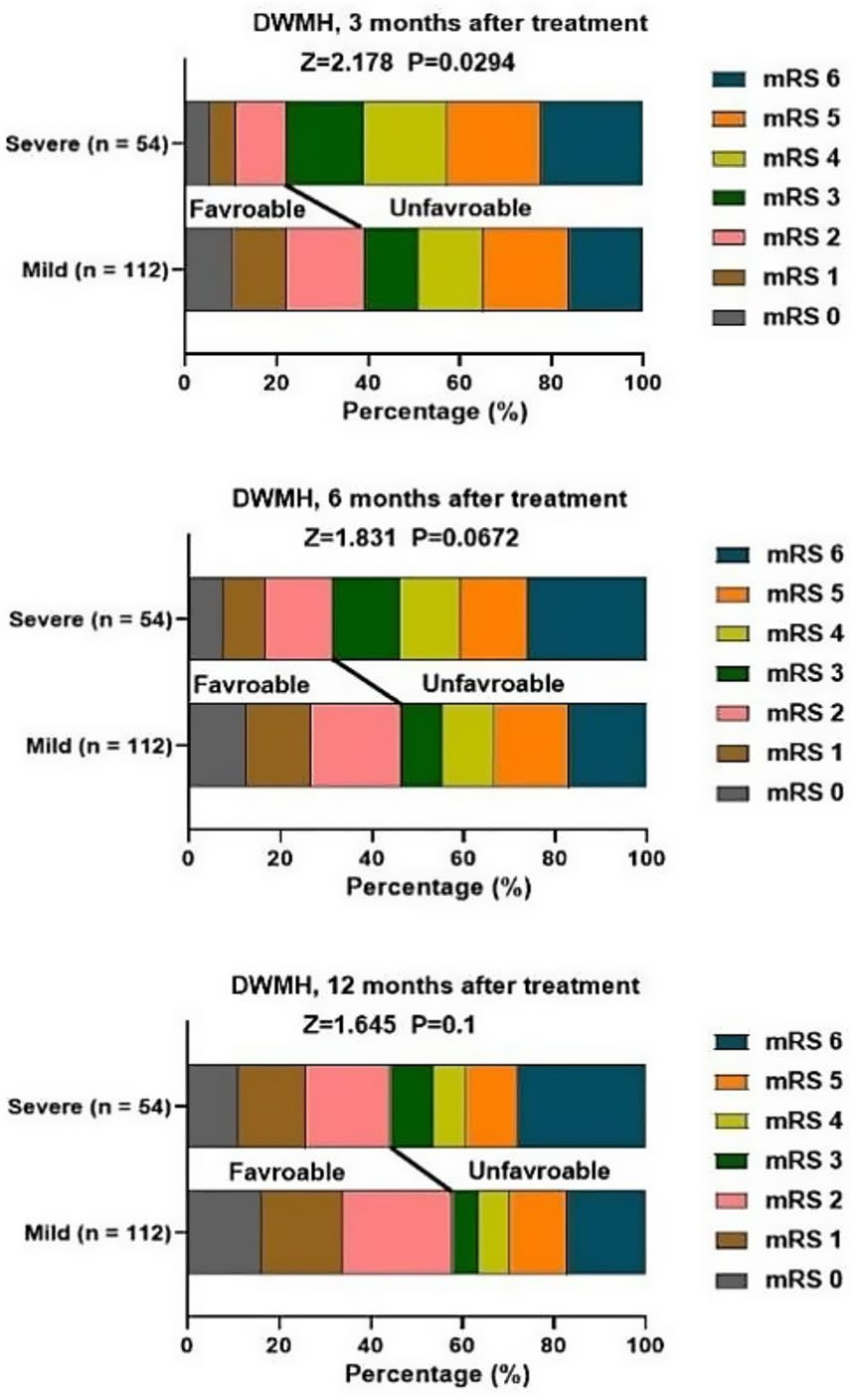
Association of DWMH and mRS score at 3, 6, 12 months after treatment.

At 3, 6, 12 months after treatment, severe PVH was significantly associated with unfavorable function outcomes of patients with stroke (Figure 2). Severe DWMH was significantly associated with unfavorable function outcomes at 3 months after treatment, but not at 6 months or 12 months after treatment (Figure 3).

### Association of WMH and neurological recovery

During the hospitalization period, the recovery of the patients’ neurological functions was evaluated (Table 3). For the PVH group, 34 patients showed improvement in their neurological function, which accounted for 30.36%. The proportion of patients with neurological function recovery in the severe group (15.15%) was significantly lower than that in the mild group (36.71%). Forty-two (25.3%) patients with DWMH showed neurological function recovery. In the severe group, 10 patients showed neurological function recovery (18.52%), while in the mild group, 32 patients showed neurological function recovery (28.57%), but this association was not significant (*p* = 0.163).

**Table 3:** Association of WMH and neurological recovery.

PVH, *n* = 112, improved or not	Mild group, *n* = 79	Severe group, *n* = 33	*z* value	*p* value
Yes, *n* = 34 (30.36%)	29 (36.71%)	5 (15.15%)	2.262	0.024
No, *n* = 78 (69.64%)	50 (63.29%)	28 (84.85%)		
DWMH, *n* = 166, improved or not	Mild group, n = 112	Severe group, n = 54	1.396	0.163
Yes, *n* = 42 (25.30%)	32 (28.57%)	10 (18.52%)		
No, *n* = 124 (74.70%)	80 (71.43%)	44 (81.48%)		

### Association of WMH and stroke recurrence

At 3 months and 6 months after treatment, the degree of PVH was not significantly associated with stroke recurrence. At 12 month, 14 cases (12.50%) of patients with PVH experienced recurrence. In the severe group, 8 cases (24.24%) had recurrence, and in the mild group, 6 cases (7.59%) had recurrence. At 12 months after treatment, a higher PVH level was significantly associated with stroke recurrence (*p* = 0.015, Table 4). For patients with DWMH, the proportion of cases with recurrence in the severe group was higher than that in the mild group at 3 months, 6 months, and 12 months after treatment (*p* < 0.05; Table 5).

**Table 4:** Association of PVH and stroke recurrence.

Recurrence or not	Mild group, *n* = 79	Severe group, *n* = 33	*z* value	*p* value
3 months			1.532	0.125
Yes, *n* = 5 (4.46%)	2 (2.53%)	3 (9.09%)		
No, *n* = 107 (95.54%)	77 (97.47%)	30 (90.91%)		
6 months			1.322	0.186
Yes, *n* = 8 (7.14%)	4 (5.06%)	4 (12.12%)		
No, *n* = 104 (92.86%)	75 (94.94%)	29 (87.88%)		
12 months			2.429	0.015
Yes, *n* = 14 (12.50%)	6 (7.59%)	8 (24.24%)		
No, *n* = 98 (87.50%)	73 (92.41%)	25 (75.76%)		

**Table 5:** Association of DWMH and stroke recurrence.

Recurrence or not	Mild group, *n* = 112	Severe group, *n* = 54	*z* value	*p* value
3 months			2.942	0.003
Yes, *n* = 13 (7.83%)	4 (3.57%)	9 (16.67%)		
No, *n* = 153 (92.17%)	108 (96.43%)	45 (83.33%)		
6 months			2.855	0.004
Yes, *n* = 22 (13.25%)	9 (8.04%)	13 (24.07%)		
No, *n* = 144 (86.75%)	103 (91.96%)	41 (75.93%)		
12 months			2.982	0.003
Yes, *n* = 26 (15.66%)	11 (9.82%)	15 (27.78%)		
No, *n* = 140 (84.34%)	101 (90.18%)	39 (72.22%)		

### Association of WMH and stroke-related death

At 3 months, 6 months and 12 months after treatment, a total of 153 patients died. The causes included brain herniation due to high intracranial pressure, secondary complications (such as aspiration pneumonia, sepsis), and malignant brain edema and hemorrhagic transformation. The proportion of deaths due to stroke in the severe PVH group was higher than that in the mild PVH group (*p* < 0.05; Table 6). There was no significant association between the severity of DWMH and stroke-related deaths at 3 months, 6 months and 12 months after treatment (*p* > 0.05; Table 7).

**Table 6:** Association of PVH and death after treatment.

Survival or not	Mild group, *n* = 79	Severe group, *n* = 33	*z* value	*p* value
3 months			1.728	0.084
Yes, *n* = 95 (84.82%)	70 (88.61%)	25 (75.76%)		
No, *n* = 17 (15.18%)	9 (11.39%)	8 (24.24%)		
6 months			2.089	0.037
Yes, *n* = 94 (83.93%)	70 (88.61%)	24 (72.73%)		
No, *n* = 18 (16.07%)	9 (11.39%)	9 (27.27%)		
12 months			2.556	0.011
Yes, *n* = 91 (81.25%)	69 (87.34%)	22 (66.67%)		
No, *n* = 21 (18.75%)	10 (12.66%)	11 (33.33%)		

**Table 7:** Association of DWMH and death after treatment.

Survival or not	Mild group, *n* = 112	Severe group, *n* = 54	*z* value	*p* value
3 months			0.965	0.335
Yes, *n* = 136 (81.93%)	94 (83.93%)	42 (77.78%)		
No, *n* = 30 (18.07%)	18 (16.07%)	12 (22.22%)		
6 months			1.355	0.175
Yes, *n* = 133 (80.12%)	93 (83.04%)	40 (74.07%)		
No, *n* = 33 (19.88%)	19 (16.96%)	14 (25.93%)		
12 months			1.617	0.106
Yes, *n* = 132 (79.52%)	93 (83.04%)	39 (72.22%)		
No, *n* = 34 (20.48%)	19 (16.96%)	15 (27.78%)		

## Discussion

In our study, we analyzed the significant prognostic value of WMH subtypes in ischemic stroke, and found that WMH subtypes were significantly related to long-term functional outcomes, recurrence, and mortality of patients with ischemic stroke. Consistently, Sun et al. indicated that a quantitative assessment of WMH provided a valuable neuro-imaging tool for enhancing the prediction of ischemic stroke recurrence risk (15). Liou et al. proposed that unfavorable functional stroke outcome was associated with MRI WMH (16). Moreover, in a RUN DMC study, it has been reported WMH is a main predictor for the development of incident dementia at 5-year follow-up in elderly with cerebral small vessel disease (17). In addition, in a LADIS study, WMH was discovered to be independently associated with general cognitive function in a sample of independently living elderly (18).

At present, the formation mechanism of WMH is unclear. Freeze et al. (19) used dynamic contrast enhanced MRI to analyze the blood–brain barrier leakage in 80 older subjects and found that the degree of leakage was positively correlated with the WMH volume and negatively correlated with the information processing speed. This suggested that the blood–brain barrier permeability was increased in the WMH region, allowing harmful substances to enter the ventricles and brain parenchyma and damaging nerve fibers (20). The blood supply to the white matter regions of the brain mainly originates from the deep perforating arteries of the deep cerebral white matter, and long-term chronic ischemia and hypoperfusion may lead to ischemic hypoxic injury of brain tissue, accompanied by corresponding pathological changes (21). Bernbaum et al. (22) proposed that chronic low cerebral blood flow was an independent risk factor for WMHs in the brain. When cerebral blood flow increases by 1 mL/100 g per minute, the risk of new WMH can be reduced by 61%, suggesting that low perfusion plays an important role in the formation of WMHs. In addition, inflammatory response, venous collagen deposition, genetics and other mechanisms may also be related to the occurrence of WMHs (23). Rastogi et al. explored the emerging role of white matter lesions in cerebrovascular disease, and indicated the pathophysiology of white matter lesions of presumed vascular origin, including the role of blood–brain barrier dysfunction, reduced cerebral blood flow and cerebrovascular reactivity, venous impairment and microembolization (24).

The histopathological results of the autopsy have shown that the ependyma in PWH region is discontinuous, and the loss of myelin was more obvious, with more severe gliosis and loss of white matter fibers; while in the DWMH area, there are more axonal injuries, more severe vacuolation and tissue damage, indicating that in addition to demyelination and gliosis, the DWMH area also have infarction changes (25). The lateral ventricular mass is located in the watershed area and is mainly supplied by a longer perforating branch, while the deep white matter of the brain is supplied by shorter branches. When the cerebral blood circulation is impaired, the former is more prone to ischemic and hypoxic changes (26).

Although our findings revealed that gender was not a relevant factor for causing stroke, a previous study has demonstrated that male patients with acute stroke and PVH are more likely to develop post-stroke depression 3 months later (27). The possible reason for this difference might be the difference in the study period, study area, and sample size used. Both PVH and DWMH were associated with functional outcomes, and their assessment criteria were based on the mRS score. We revealed that severe DWMH was only associated with unfavorable functional outcomes only at 3 months after treatment. In contrast, severe PVH was associated with unfavorable functional outcomes of stroke at 3, 6, and 12 months after treatment. Besides, mild PVH rather than DWMH was significantly associated with neurological recovery. The reasons are as follows: (1) PVH is located in the area of high-density long-associated white matter fiber tracts, which connect to the widely distributed cortical regions that support various cognitive functions. Therefore, it has a closer relationship with cognitive functions (28). (2) The relationship between PVH and early cognitive impairment after stroke due to cerebral small vessel disease is more significant. The vascular lesions and hemodynamic changes caused by cerebral small vessel disease are more pronounced in the periventricular area, making the nerve fibers in this area more susceptible to damage and thereby affecting cognitive function (29). Consistently, Liu et al. suggested that PVH was associated with poor functional prognosis in stroke patients within the first month after the onset of the disease, while DWMH was not related to this (16). Moreover, mild PVH was significantly associated with neurological recovery, suggesting PVH may be associated with neuroplasticity, and this neuroplasticity may result from adaptive changes in neuronal activity or regulation of synaptic transmission efficiency, which may open up a way for targeted rehabilitation treatment, such as balance and gait training and walking training.

Our study indicated that higher DWMH was significantly associated with stroke recurrence at 3, 6, 12 months after treatment, while higher PVH was only significantly associated with stroke recurrence at 12 months after treatment. The reason are as follows: (1) DWMH are directly associated with small vessel lesions such as lacunar infarction, and these lesions are the core pathological basis for the recurrence of ischemic stroke (30). (2) The DWMH is caused by insufficient blood supply from the arteries. The branches such as the lentiform arteries that originate from the main trunk of the cerebral artery supply blood to the DWMH. When these arteries are diseased, the deep cerebral white matter is more susceptible to ischemic damage. Therefore, the DWMH can more directly reflect the severity of small vessel lesions, thereby improving the accuracy of recurrence prediction. (31). (3) The DWMH have a stronger association with cerebral small vessel diseases (such as atherosclerosis and fibro-atheromatous changes). These lesions directly increase the risk of recurrence of ischemic events by affecting the integrity of the vascular walls (32). Similarly, Xu et al. indicated that severe PVH was associated with stroke recurrence during a median follow-up of 4.9 years (33). PVH is particularly associated with acceleration of functional decline (34). A type of integrated WMH is associated with a higher risk of mortality and ischemic stroke (35). However, our study indicated that at 3 months, 6 months and 12 months after treatment, the proportion of deaths due to stroke in the severe PVH group was higher than that in the mild PVH group. There was no significant association between the severity of DWMH and stroke-related deaths at 3 months, 6 months and 12 months after treatment. The severity of leukoaraiosis has a significant impact on the prognosis of acute ischemic stroke patients after reperfusion therapy, mainly manifested in functional prognosis, complication risk and mortality rate. A recent meta-analysis involving 6,460 patients has demonstrated that moderate-to-severe leukoaraiosis on baseline imaging are likely to have worse clinical and safety outcomes after reperfusion therapy (36).

Our research has some limitations. First, this study is a retrospective study, and its inherent biases can have an impact on the prognosis. Second, patients who did not undergo MRI examinations were excluded from the study, but the clinical outcomes of these patients are unknown, and there may be unobserved clinical differences. Third, lacunar stroke is a sign of cerebral small vessel disease and accounts for 25% of ischemic strokes, and the pathophysiology, prognosis, and clinical features of lacunar strokes are different from other acute cerebrovascular diseases (37), but the differences between lacunar and non-lacunar infarction in our study were not analyzed. In addition, a clinical report has showed that cerebral infarcts in the territory of the posterior cerebral artery have a better prognosis than infarcts in the territory of the middle cerebral artery (38). However, in our study, we did not analyze the differences between cerebral infarction in the posterior cerebral artery region and that in the middle cerebral artery region. Fourth, the impact of stroke subtypes (such as lacunar and non-lacunar, thrombotic and thromboembolic) on white matter hyperintensities (PVH and DWMH) were not stratified. Since these causes have different pathophysiological mechanisms and prognoses, this may confound the association between white matter hyperintensities and outcomes. Furthermore, although our research indicates a significant correlation between stroke-related deaths and pulmonary vascular hypertension, it did not explore possible contributing factors such as weakness, infection, or dysphagia. Therefore, in the future, we will conduct more in-depth and larger-scale research to further address the above questions.

## Conclusion

Our study indicates that PVH and DWMH subtypes were significantly related to long-term functional outcomes, recurrence, and mortality of patients with ischemic stroke, suggesting that prognostic value of PVH and DWMH subtypes in ischemic stroke.

## Data Availability

The original contributions presented in the study are included in the article/supplementary material, further inquiries can be directed to the corresponding author.
